# Higher-order topology induced by structural buckling

**DOI:** 10.1093/nsr/nwab170

**Published:** 2021-09-09

**Authors:** Huaqing Huang, Feng Liu

**Affiliations:** School of Physics, Peking University, Beijing 100871, China; Collaborative Innovation Center of Quantum Matter, Beijing 100871, China; Center for High Energy Physics, Peking University, Beijing 100871, China; Department of Materials Science and Engineering, University of Utah, Salt Lake City, UT 84112, USA

**Keywords:** higher-order topological insulators, structural buckling, rotation-reflection symmetry, buckled honeycomb antimony monolayer

## Abstract

Higher-order topological insulator (HOTI) states, such as two-dimension (2D) HOTI featured with topologically protected corner modes at the intersection of two gapped crystalline boundaries, have attracted much recent interest. However, the physical mechanism underlying the formation of HOTI states is not fully understood, which has hindered our fundamental understanding and discovery of HOTI materials. Here we propose a mechanistic approach to induce higher-order topological phases via structural buckling of 2D topological crystalline insulators (TCIs). While in-plane mirror symmetry is broken by structural buckling, which destroys the TCI state, the combination of mirror and rotation symmetry is preserved in the buckled system, which gives rise to the HOTI state. We demonstrate that this approach is generally applicable to various 2D lattices with different symmetries and buckling patterns, opening a horizon of possible materials to realize 2D HOTIs. The HOTIs so generated are also shown to be robust against buckling height fluctuation and in-plane displacement. A concrete example is given for the buckled $\beta $-Sb monolayer from first-principles calculations. Our finding not only enriches our fundamental understanding of higher-order topology, but also opens a new route to discovering HOTI materials.

## Teaser

An effective approach of structural buckling is proposed to induce higher-order topological phase in various 2D materials with different symmetries and buckling patterns, opening a horizon of possible materials to realize 2D HOTIs.

## INTRODUCTION

The discovery of topological insulators (TIs) [1,2] has inspired extensive exploration of other novel topological states, such as the TCIs [3,4], and more recently HOTIs [5–18]. In general, the characteristic properties of these topological states are well understood. The TIs and TCIs have a gapped *d*-dimensional bulk and topologically protected gapless states on *d-*1 dimensional boundaries, while the HOTIs (*n*-th order TIs with $1 < n \le d$) have a similar gapped bulk, but the gapless states emerge not at *d-*1 but at lower *d-n* dimensions. For example, a second-order HOTI in 2D hosts topological states located at its 0D corners between distinct gapped 1D edges. Moreover, the physical mechanism underlying the formation of TIs and TCIs is also well understood, and generally involves a band inversion process. Accordingly, abundant TI/TCI materials have been discovered and/or proposed based on this mechanism, through band inversions induced by, for example lattice/orbital symmetry [19–21], quantum-well structure [22], strain [23] and surface adsorption/growth [24,25], etc. On the contrary, the physical mechanism underlying the formation of HOTIs is less clear. This knowledge gap has not only lessened our fundamental understanding of HOTI states, but also inevitably hindered our ability to discover HOTI materials. So far, only very few candidate materials have been theoretically proposed to host 2D HOTI states, including phosphorene [26], graph(di)yne [27–29] and twisted bilayer graphene or boron nitride at special angles [30,31].

In this work, we reveal a generic physical mechanism of transforming a 2D TCI state into a HOTI state via structural buckling. It has been previously shown that structural buckling provides a key degree of freedom to tailor materials’ properties [32], such as thermal conductivity [33], magnetic response [34] and spin-orbit interaction [35]. Structural buckling can also facilitate the well-known band inversion mechanism to induce topological transition of TI states [24,36,37]. But our finding here is mechanistically different. In general, structural buckling, which breaks the in-plane mirror symmetry (${M_z}$), would destroy the ${M_z}$-protected TCI states in 2D planar lattices. However, we realize that topological gapless states of TCIs are gapped out differently between adjacent edges of different orientations subject to the remaining combination of mirror and rotation symmetry (${S_n} = {M_z}{C_n}$). Consequently, a HOTI with topological corner states will emerge ubiquitously. Moreover, we found that even an approximate ${S_n}$ symmetry suffices for the existence of HOTI: the 0D topological corner states are robust against buckling height fluctuation and symmetry breaking perturbations. We further demonstrate that this newly discovered structural buckling approach is generally applicable to various lattices with different symmetry and buckling patterns, which greatly extends possible material choices to realize HOTIs. Finally, we calculate from first-principles the HOTI state in the buckled β-Sb honeycomb monolayer as a concrete material example.

## RESULTS

### HOTI in a buckled square lattice

**Figure 1.**
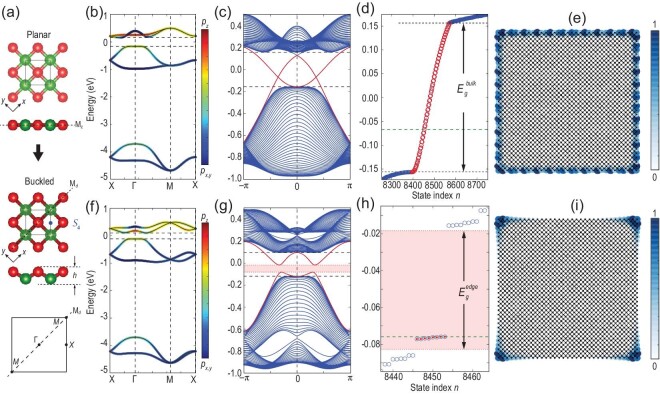
Comparison of electronic structures between planar and buckled square lattices. (a) Illustration of structural buckling. The color (red/green) marks atomic buckling direction (upwards/downwards). The buckling height is $h= 0.2a$, with $a$ being the bond length. Bulk band structures of (b) the planar and (f) buckled square lattices. For comparison, both band structures are calculated using two-atom unitcells. The parameters used here are ${\varepsilon _{x,y}} = - 1.88,{\varepsilon _z} = - 0.88,{V_{pp\sigma }} = 0.5,{V_{pp\pi }} = - 0.15$, and $\lambda = {\rm{}}1.25$ eV. Band structure of nanoribbons of (c) the planar and (g) buckled square lattices. Energy spectrum of square nanodisks of (d) the planar and (h) buckled square lattices. Spatial intensity distribution |*ψ*(**r**)|^2^ of topologically protected (e) edge states of the planar square lattice and (i) corner modes of the buckled square lattice.

**Figure 2.**
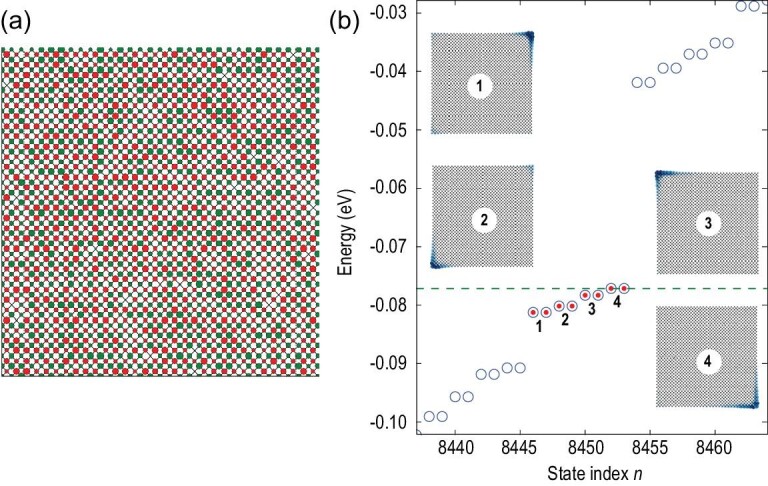
(a) A buckled square lattice with buckling height fluctuation. The red (green) dots represent upwards (downwards) buckled atoms, the size of dots denote random buckling amplitudes within $[ {0,0.1a} ]$, with $a$ being the bond length. (b) Energy spectrum of a square disk of the buckled square lattice with random buckling heights. Insets show the spatial intensity distribution |*ψ*(**r**)|^2^ of in-gap corner states.

To illustrate the structural buckling induced higher-order topology, we first take the mirror-protected TCI state in a square lattice as an example. Figure 1 shows the electronic structures of the planar square lattice and the buckled square lattices with buckling height $h= 0.2a$ (where $a$ is the bond length), respectively. The orbital-resolved band structures of both systems exhibit signatures of a band inversion between ${p_z}$ and ${p_{x,y}}$ orbitals around the ${\rm{\Gamma }}$ point (see Fig. 1b and f), implying their nontrivial electronic topology. To identify the TCI state in the planar square lattice, we calculated the mirror Chern number ${C_m} = 2$, which guarantees the existence of topological edge states, as displayed in Fig. 1c–e. It is noted that the bulk band structures of the planar and buckled structures are similar to each other except for slight band splitting, as shown in Fig. 1b and f. However, the edge state changes dramatically after the structural buckling. As shown in Fig. 1g, the topological edge states are clearly gapped, indicating that the TCI state is destroyed because of the structural buckling induced mirror symmetry breaking. By further studying the energy spectrum of the finite square disk for the buckled lattice, we find that there are eight states (marked as red dots) around the Fermi level within the edge-gap region (light-red area) of the buckled system, as shown in Fig. 1h. Remarkably, from spatial intensity distribution ${| {\psi ( {{\bf r}} )} |^2}$, we further find that these midgap states are localized on four corners of the sample (see Fig. 1i), which is distinct from the topologically protected extended edge states along the whole perimeter of TCIs (see Fig. 2e). This implies that the buckled system is a HOTI. As a result of the time-reversal symmetry, corner modes always appear in pairs (Kramers pair) [8]. Therefore, there are a total of eight corner modes in the disk of the buckled square lattice, which is different from previously studied spinless HOTIs [27–29].

### k · p analysis

To further verify the nontrivial higher-order topological nature, we performed *k* · *p* analysis and derived an edge theory for the buckled square lattice (see Supplementary data). First, we derived the effective Hamiltonian for the planar square lattice as

$$\begin{eqnarray*}
H &=& \left( {{m_0} - {m_1}{k^2}} \right){\sigma _z}{\tau _0} + {v_1}\!\left( {k_x^2 - k_y^2} \right){\sigma _x}{\tau _z}\nonumber\\
&&-\, {v_2}{k_x}{k_y}{\sigma _y}{\tau _0},
\end{eqnarray*}$$

where ${m_0},{m_1},{v_1}$ and ${v_2}$ are parameters. ${\tau _z} = \pm 1$ denotes two mirror sectors and ${\sigma _z} = \pm 1$ denotes basis states. As the structural buckling breaks the mirror symmetry ${M_z}$, an extra term which mixes two mirror sectors was added into the above Hamiltonian,

$$\begin{equation*}
{\rm{}}{H_b} = {v_b}{\sigma _y}\!( {{k_x}{\tau _x} + {k_y}{\tau _y}}).
\end{equation*}$$

We then derived the effective Hamiltonian of the 1D edge states with valleys at $ \pm {{\rm{k}}_0}$ along the edge

$$\begin{equation*}
{\rm{}}{H_{\textit{edge}}} = v\!\left( {k \pm {k_0}} \right)\!{s_z} \pm {m_b}{s_y},
\end{equation*}$$

where $v$ is the velocity of the edge states, ${s_{x,y,z}}$ is Pauli matrix and $k$ is the momentum in the 1D Brillouin zone of the edge. The last term is the structural buckling induced mass term which gaps out the topological Dirac edge states. The 1D massive Dirac edge spectrum admits a ${{\rm{Z}}_2}$ classification depending on the sign of the mass term. As the structural buckling breaks ${M_z}$ but preserves ${S_4} = {M_z}{C_4}$ symmetry, the mass term changes sign alternatively between adjacent edges following the ${S_4}$ symmetry in the buckled square lattice. Consequently, 0D corner states arise as the topological domain-wall state between two edges belonging to distinct topological classes according to the Jackiw-Rebbi mechanism [38]. Furthermore, the mass term is odd under the vertical diagonal mirror symmetry ${M_d}$ or ${M_{\bar{d}}}$, which guarantees the emergence of topological corner states at the intersection of two edges related by the diagonal mirror symmetry.

Alternatively, an intuitive argument of the structural buckling induced nontrivial higher-order topology can be made by utilizing the vertical mirror symmetry, following the approach by Langbehn *et al.* [7]. As the buckled square lattice is mirror symmetric under ${M_d}$ (see Fig. 1a), one can divide the wave functions in the intersection line (M-${\rm{\Gamma }}$-M) between the diagonal mirror plane and the 2D Brillouin zone into two separate sets with opposite ${M_d}$ eigenvalues ($ \pm i$). For each set, one can evaluate its Zak phase [39] through the cell-periodic Bloch function ${u_{ \pm i}}( k )$:

$$\begin{equation*}
{\rm{}}{\varphi _{ \pm i}} = {\rm{}}i\oint \langle {\rm{}}{u_{ \pm i}}\!\left( k \right)\left| {{\partial _k}} \right|{u_{ \pm i}}\!\left( k \right)\rangle {\rm{}}dk,
\end{equation*}$$

which is essentially related to the mirror-grad winding number for the 1D effective Hamiltonian in the mirror-invariant line. As the Zak phase represents the electric polarization for the mirror subspace of the 1D system, the calculated Zak phase ${\varphi _{ \pm i}} = \pi $ indicates the presence of end modes at corners between two edges connected by ${M_d}$, as shown in Fig. 1i. This implies the system is a second-order TI [40].

As the chiral (sublattice) symmetry is preserved in the simplified model, the bulk band topology discussed above also suggests the coexistence of a fragile TCI phase [12], which would give rise to gapless edge states only along the smooth ${M_d}$-preserving edge. However, such a gapless edge state can be easily destroyed by breaking the sublattice symmetry such as via a staggered potential, which also breaks the ${S_4}$ symmetry. In contrast, the topological corner states are much more robust against such perturbations (see Supplementary data).

### Robustness against buckling height fluctuation

We checked the robustness of topological corner states against buckling height fluctuation. Instead of uniformly buckling with the same height, random buckling heights within $[{ - h/2,h/2} ]$ were considered in the buckled square lattice (see Fig. 2a). In comparison with Fig. 1h, the energy levels of four pairs of corner states split slightly, as shown in Fig. 2b. However, different from the case with uniform buckling height, the wavefunctions of the corner states are asymmetric among four corners, that is each pair of corner states mainly localized on one corner (see insets of Fig. 2b). Therefore, these in-gap topological corner states are rather robust against random buckling height fluctuation. In addition, we also found that topological corner states are robust against weak in-plane random displacements (see Supplementary data). This confirms that as long as the ${S_4}$ symmetry is roughly overall preserved, the topological corner state is preserved, which greatly eases its experimental realization.

### HOTIs in other buckled lattices

**Figure 3.**
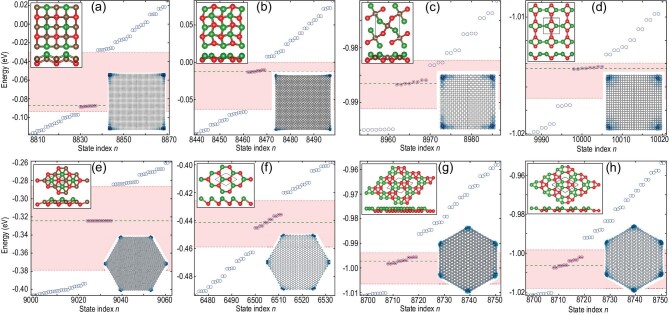
HOTIs in other lattices with structural buckling. Energy spectrum of finite nanodisks of (a) FeSe-type tetrahedral-buckled square lattice, (b) buckled snub square lattice, (c) distorted Lieb lattice, (d) truncated square lattice, (e) octahedral-buckled trigonal lattice, (f) buckled honeycomb lattice, (g) buckled snub hexagonal lattice, (h) buckled ruby lattice. The parameters are presented in Supplementary data. Left inset in each panel shows the top and side views of lattice structures where the color (green/red) marks atomic buckling direction (upwards/downwards). Right inset in each panel shows the spatial distribution |*ψ*(**r**)|^2^ of topologically protected corner modes.

In addition to the case study discussed above, we also considered other lattices with different buckling forms. Notably, we considered another buckled square lattice with a FeSe-type tetrahedral buckling which consists of four atoms per unitcell (see left inset of Fig. 3a). As the ${S_4}$ symmetry is retained in the tetrahedral-buckled square lattice, this system is also a HOTI, which is characterized by the existence of eight corner modes around the Fermi level in the energy spectrum of its square nanodisk, as shown in Fig. 3a. This indicates that the physical mechanism of realizing HOTIs via structural buckling also works in square lattices with different buckling forms. In fact, it is expected to be generally applicable to other lattices with different symmetries. To confirm this, we further investigated the buckled snub square, distorted Lieb, truncated square lattices with ${S_4}$ symmetry as well as the buckled trigonal, honeycomb, ruby, and snub hexagonal lattices with ${S_6}$ symmetry (see Supplementary data for more details). According to the energy spectrum analysis, the square (hexagonal) nanodisks support eight (12) corner states around the Fermi level, as shown in Fig. 3b–h. The spatial distribution of these states also clearly demonstrates that they are localized at corners of nanodisks (see insets of Fig. 3). Because of the finite-size-effect-induced weak coupling between adjacent corners, there exists slight energy splitting for these corner states around the Fermi level, which is exponentially suppressed with the increasing nanodisk size. Overall, the structural-buckling mechanism is general and is applicable to various systems with different symmetries and buckling patterns. Given that various 2D TCIs have been found previously, our proposed approach greatly extends the range of candidate materials for realizing HOTIs.

### Material example

**Figure 4.**
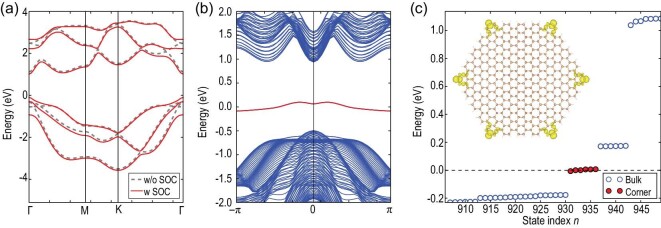
HOTI in the buckled *β*-Sb honeycomb monolayer. (a) Bulk band structures of the buckled honeycomb lattice of Sb without (gray dashed line) and with SOC (red solid line). As the effect of SOC is weak according to the bulk bands, the calculations of nanoribbons and nanodisks are performed without SOC. (b) Band structure of a nanoribbon of the buckled Sb monolayer without SOC. (c) Energy spectrum of a hexagonal-shaped nanodisk with H-saturated edges for the buckled Sb monolayer. The inset shows the real-space charge distribution of corner states around the Fermi level.

Finally, we took the $\beta $-Sb monolayer as a concrete material manifestation. A $\beta $-Sb monolayer that has been experimentally synthesized [41–44], crystallizes in a buckled honeycomb lattice with two Sb atoms per unitcell. The naturally buckled Sb honeycomb lattice was previously found to be a trivial insulator, but would become a mirror-protected TCI in its planar structure under an in-plane tensile strain [37]. Here we show that a variety of topological phases can be realized in the Sb monolayer by varying the degree of structural buckling. Based on first-principles calculations, we found a HOTI state in the natively buckled $\beta $-Sb monolayer. As shown in Fig. 4a, the buckled Sb honeycomb lattice is an insulator with an energy gap of $ \sim $1.1 eV. As the effect of the spin-orbit coupling (SOC) on bulk band structures is weak, hereafter we performed calculations of nanoribbons and nanodisks without SOC, unless otherwise specified. Interestingly, we found a flat edge state in the energy gap of nanoribbons (see Fig. 4b), implying a possible topological effect. To further identify its higher-order topology, we calculated a hexagonal-shaped nanodisk of the buckled Sb honeycomb lattice. Evidently, there are six (12 if spin is counted) states around the Fermi level and these states are localized at corners as shown in Fig. 4c, confirming the existence of topological corner states. Moreover, by scanning the evolution of band topology with the buckling height, we found a quantum spin Hall state in the intermediate region between the planar TCI phase and the natively buckled HOTI phase (see Supplementary data). This indicates that the structural buckling significantly affects the band topology of the Sb monolayer. As differently buckled Sb monolayers can be epitaxially grown on various substrates [42–45] and/or controlled by external strains, one, therefore, expects to observe rich topological physics in Sb monolayers with tunable structural buckling.

## CONCLUSION

In conclusion, we have revealed a generic physical mechanism of structural buckling underlying the transition from the mirror-protected TCI to HOTI state in 2D materials. The topological corner states of the HOTIs are robust against buckling height fluctuation and similar HOTIs are demonstrated in various buckled lattices with ${S_4}$ or ${S_6}$ symmetry. By taking advantage of the broad material categories of previously studied 2D TCIs, such as films of SnTe family compounds [46], our finding opens a new route towards discovering HOTIs with a wealth of possibilities, which is expected to draw immediate experimental attention. For example, new candidate materials of HOTIs with similar buckled structures are expected to be predicted by utilizing the high-throughput computation of 2D materials. The structural buckling mechanism may also work in 3D to stimulate the realization of 3D HOTIs. For example, by stacking these 2D HOTIs with interlayer coupling to form 3D HOTIs, or by applying strains to realize HOTIs via lateral lattice expansion accompanied with buckling reduction [47] and structural distortions in 3D materials [9]. Our discovery may also shed light on the exploration of higher-order topology in other fields such as phononic, photonic, microwave and electrical circuit systems.

Note: After submission, we become aware of another work [15] studying the higher-order topological phase in buckled group-V honeycomb lattices.

## METHODS

### Tight-binding model

We considered a general tight-binding model for 2D lattices with three $p$ orbitals per site. The Hamiltonian is given by

$$\begin{eqnarray*}
H&=& \mathop \sum \limits_{i,\mu } {{\rm{\varepsilon }}_{\rm{\mu }}}{{\bf c}}_{i\mu }^\dagger \cdot {\rm{}}{{{\bf c}}_{i\mu }}{\rm{}} + \mathop \sum \limits_{\langle i,j\rangle ,\mu } {{\bf c}}_{i\mu }^\dagger {{{\bf T}}_{i,j}}{\rm{}}{{{\bf c}}_{j\mu }}\nonumber\\
&& +\, i{\rm{\lambda }}\mathop \sum \limits_{i,\mu \nu } \left( {{{\bf c}}_{i\mu }^\dagger \times {{{\bf c}}_{i\nu }}} \right) \cdot {{{\bf s}}_{\mu \nu }},
\end{eqnarray*}$$

where ${{\bf c}}_{i\mu }^\dagger = {( {c_{i{p_x}}^\dagger ,c_{i{p_y}}^\dagger ,c_{i{p_z}}^\dagger } )_\mu }$ and ${{{\bf c}}_{i\mu }} = ( {{c_{i{p_x}}},{\boldsymbol{}}{c_{i{p_y}}},{\boldsymbol{}}{c_{i{p_z}}}} )_\mu ^T{\boldsymbol{}}$ are electron creation and annihilation operators with spin $\mu {\rm{}}( {{\rm{}} = {\rm{}} \uparrow ,{\rm{}} \downarrow } )$ at the *i*-th site, respectively. ${\varepsilon _\mu } = {( {{\varepsilon _x},{\varepsilon _y},{\varepsilon _z}} )_\mu }$ are the on-site energies for the three *p* orbitals. ${\rm{\lambda }}$ is the spin-orbit coupling (SOC) strength and ${{\bf s}}= {\rm{}}( {{\sigma _x},{\sigma _y},{\sigma _z}} )$ are the Pauli matrices. ${{{\bf T}}_{i,j}} = {\rm{}}{[ {{t_{\alpha \beta }}( {{{{\bf r}}_{ij}}} )} ]_{3 \times 3}}$ is a $3 \times 3$ matrix containing Slater-Koster hopping integrals ${t_{\alpha \beta }}( {{{{\bf r}}_{ij}}} ),$ which depends on the orbital type $( {\alpha ,\beta = {p_x},{p_y},{p_z}} )$ and the intersite vector ${{{\bf r}}_{ij}}$ from site *i* to *j*. Previously, it was known that by considering a band inversion between ${p_{x,y}}$ and ${p_z}$ orbitals, mirror-protected TCI states could be realized in various 2D planar lattices [48]. Here we illustrate that by structural buckling these TCIs can be intriguingly driven into HOTIs.

### DFT calculation

The first-principles calculations were performed within the framework of density functional theory using the Vienna *ab initio* simulation package [49] with the Perdew-Burke-Ernzerhof-type generalized gradient approximation [50] in the projector augmented wave method. A default kinetic energy cutoff is adopted in all calculations. A $30 \times 30 \times 1$, $10 \times 1 \times 1$ and single ${\rm{\Gamma }}$-centered **k**-mesh of the Brillouin zone sampling are used for the bulk, nanoribbon and nanodisk calculations, respectively. The lattice constant for the buckled Sb honeycomb lattices is 4.21 Å, and the buckling height is 1.6 Å. Nanoribbons with $ \sim $80 atoms per unitcell and nanodisks with $ \sim $400 atoms are calculated to show the edge states and corner states, respectively.

## Supplementary Material

nwab170_Supplemental_File
