# Supplementary material for: Higher-order topology induced by structural buckling
Source: Natl Sci Rev. 2021 Sep 9;9(8):nwab170. doi: 10.1093/nsr/nwab170 (PMC9450183; doi:10.1093/nsr/nwab170)
Supplement: nwab170_Supplemental_File [file nwab170_supplemental_file.pdf]

Supplemental Material for "Structural buckling induced higher-order topology"

Huaqing Huang[a1,2,3] and Feng Liu[b4]

[1] *School of Physics, Peking University, Beijing 100871, China*
[2] *Collaborative Innovation Center of Quantum Matter, Beijing 100871, China*
[3] *Center for High Energy Physics, Peking University, Beijing 100871, China*
[4] *Department of Materials Science and Engineering, University of Utah, Salt Lake City, Utah 84112, USA*

(Dated: August 4, 2021)

[a] Corresponding author: huaqing.huang@pku.edu.cn
[b] Corresponding author: fliu@eng.utah.edu

CONTENTS

I. Tight-binding model for square lattice 2

II. fragile TCI in buckled square lattice 4

III. Robustness of topological corner states against staggered potentials 4

IV. Robustness against buckling height fluctuation 5

V. Robustness against random in-plane displacement 5

VI. tight-binding model parameters for various lattices with different symmetries 5

VII. DFT calculation of buckled Sb honeycomb lattices 5

References 6

I. TIGHT-BINDING MODEL FOR SQUARE LATTICE

We consider a general tight-binding model with three orbitals (p_x, p_y, p_z) per site in a square lattice. The Hamiltonian is given by

$$H = H_0 + H_1 + H_2 + H_{soc}, \tag{1}$$

$$H_0 = \begin{pmatrix} \epsilon_x & 0 & 0 \\ 0 & \epsilon_y & 0 \\ 0 & 0 & \epsilon_z \end{pmatrix}, \tag{2}$$

$$H_1 = \begin{pmatrix} 2V_\sigma \cos k_x + 2V_\pi \cos k_y & 0 & 0 \\ 0 & 2V_\sigma \cos k_y + 2V_\pi \cos k_x & 0 \\ 0 & 0 & 2V_\pi(\cos k_y + \cos k_x) \end{pmatrix}, \tag{3}$$

$$H_2 = \begin{pmatrix} 2(V_\sigma + V\pi)\cos k_x \cos k_y & -2(V\sigma - V\pi)\sin k_x \sin k_y & 0 \\ -2(V\sigma - V\pi)\sin k_x \sin k_y & 2(V\sigma + V\pi)\cos k_x \cos k_y & 0 \\ 0 & 0 & 4V\pi \cos k_x \cos k_y \end{pmatrix}, \tag{4}$$

$$H_{soc} = \lambda \begin{pmatrix} 0 & -is_z & is_y \\ is_z & 0 & -is_x \\ -is_y & is_x & 0 \end{pmatrix}, \tag{5}$$

$$\tag{6}$$

where s_x, s_y, s_z are Pauli matrices for spin.

It is well known that for a system with the $z \to -z$ mirror symmetry, the mirror operator $\mathcal{M}_z$ commutes with the Hamiltonian: $[H, \mathcal{M}_z] = 0$. Therefore, one can divide the Hamiltonian into two blocks based on the mirror symmetry. The mirror operator $\mathcal{M}_z$ for two-dimensional systems is given by

$$M_z = \begin{pmatrix} s_z & 0 & 0 \\ 0 & s_z & 0 \\ 0 & 0 & -s_z \end{pmatrix}. \tag{7}$$

Accordingly, the Hamiltonian H is divided as

$$H_m = U_z^\dagger H U_z = \begin{pmatrix} H_{-i} & 0 \\ 0 & H_{+i} \end{pmatrix}, \tag{8}$$

$$H_{\pm i} = \begin{pmatrix} H_z & i\lambda & \pm\lambda \\ -i\lambda & H_y & \pm i\lambda + H_{xy} \\ \pm\lambda & \mp i\lambda + H_{xy} & H_x \end{pmatrix}, \tag{9}$$

$$H_z = \epsilon_z + 2V_\pi(\cos k_x + \cos k_y) + 4V_\pi \cos k_x \cos k_y, \tag{10}$$

$$H_y = \epsilon_y + 2V_\sigma \cos k_y + 2V_\pi \cos k_x + 2(V_\pi + V_\sigma)\cos k_x \cos k_y, \tag{11}$$

$$H_x = \epsilon_x + 2V_\sigma \cos k_x + 2V_\pi \cos k_y + 2(V_\pi + V_\sigma)\cos k_x \cos k_y, \tag{12}$$

$$H_{xy} = -2(V_\sigma - V_\pi)\sin k_x \sin k_y. \tag{13}$$

Taking the $-i$ sector as an example, one can further change the basis to $\{p_z, p_+ = \frac{1}{\sqrt{2}}(p_x + ip_y), p_- = \frac{1}{\sqrt{2}}(p_x - ip_y)\}$, and the Hamiltonian becomes

$$\tilde{H}_{-i} = \begin{pmatrix} 1/2(H_x + H_y) - \lambda & 1/2(H_x - H_y) - iH_{xy} & -\sqrt{2}\lambda \\ 1/2(H_x - H_y) + iH_{xy} & 1/2(H_x + H_y) + \lambda & 0 \\ -\sqrt{2}\lambda & 0 & H_z \end{pmatrix}. \tag{14}$$

Then, one obtains an effective 2×2 Hamiltonian for the two bands around the Fermi level using the downfolding technique and expands the Hamiltonian around Γ point up to to k-quadratic order,

$$H_{-i}^{\text{eff}} = \begin{pmatrix} M_0 - C_0(k_x^2 + k_y^2) & C_2(k_x^2 - k_y^2) + iC_3 k_x k_y \\ C_2(k_x^2 - k_y^2) - iC_3 k_x k_y & M_1 - C_1(k_x^2 + k_y^2) \end{pmatrix}, \tag{15}$$

where M_0, M_1, C_0, C_1, C_2, and C_3 are parameters. The effective Hamiltonian can be expressed as $H_{-i}^{\text{eff}} = d_0\sigma_0 + \mathbf{d}\cdot\boldsymbol{\sigma}$ with $\boldsymbol{\sigma} = (\sigma_x, \sigma_y, \sigma_z)$ being Pauli matrices. Using the $\mathbf{d}$ vector, the Chern number is defined as

$$C_{-i} = \frac{1}{4\pi}\iint dk_x dk_y \hat{\mathbf{d}} \cdot (\frac{\partial \hat{\mathbf{d}}}{\partial k_x} \times \frac{\partial \hat{\mathbf{d}}}{\partial k_y}), \tag{16}$$

where $\hat{\mathbf{d}} = \mathbf{d}/|\mathbf{d}|$. Accordingly, we have found that $C_{-i} = 2$, implying that the planar square lattice is a TCI.

Similar derivation is also applicable for the other mirror sector which gives rise to the effective Hamiltonian H_{+i}^{eff}:

$$H_{+i}^{\text{eff}} = \begin{pmatrix} M_0 - C_0(k_x^2 + k_y^2) & -C_2(k_x^2 - k_y^2) + iC_3 k_x k_y \\ -C_2(k_x^2 - k_y^2) - iC_3 k_x k_y & M_1 - C_1(k_x^2 + k_y^2) \end{pmatrix}. \tag{17}$$

For convenience, we then simplify the effective TCI Hamiltonian based on the basis set $\{|p_z, \uparrow\rangle, |p_-, \downarrow\rangle, |p_z, \downarrow\rangle, |p_+, \uparrow\rangle\}$

$$H^{\text{eff}} = (m_0 - m_1 k^2)\sigma_z\tau_0 + v_1(k_x^2 - k_y^2)\sigma_x\tau_z - v_2 k_x k_y \sigma_y \tau_0, \tag{18}$$

where σ_i and τ_j $(i, j = 0, x, y, z)$are Pauli matrices. m_0, m_1, v_1 and v_2 are parameters and $k^2 = k_x^2 + k_y^2$. The above Hamiltonian can also be derived from the theory of invariant by considering the symmetries of the system[1].

Due to structural buckling, the mirror symmetry $\mathcal{M}_z$ is broken, extra terms which mix two mirror sectors appear in the original Hamiltonian. By performing similar downfolding procedure, an structural buckling induced extra term emerges in Eq. (18):

$$H_b = v_b \sigma_y (k_x \tau_x + k_y \tau_y). \tag{19}$$

Then, the effective Hamiltonian for the buckled square lattice reads

$$H_b^{\text{eff}} = H^{\text{eff}} + H_b. \tag{20}$$

Having above Hamiltonian, one can then derive an effective model for different edges. For example, let us consider a flat edge at $x = 0$ with the square lattice occupying $x < 0$. The edge states are solved from

$$H_{-i}^{\text{eff}}(k_x \to -i\partial_x, k_y)\psi = E\psi \tag{21}$$

After some tedious algebra[2,3], we obtain the effective model for these edges, which can be described unanimously as:

$$H_{edge} = v(k \pm k_0)s_z \pm m_b s_y, \tag{22}$$

where $\pm k_0$ are the band crossing points of the gapless edge states of the planar square lattice, v is the velocity of the edge states, $s_{x,y,z}$ is Pauli matrix, and k is the momentum in the 1D Brillouin zone of the edge.

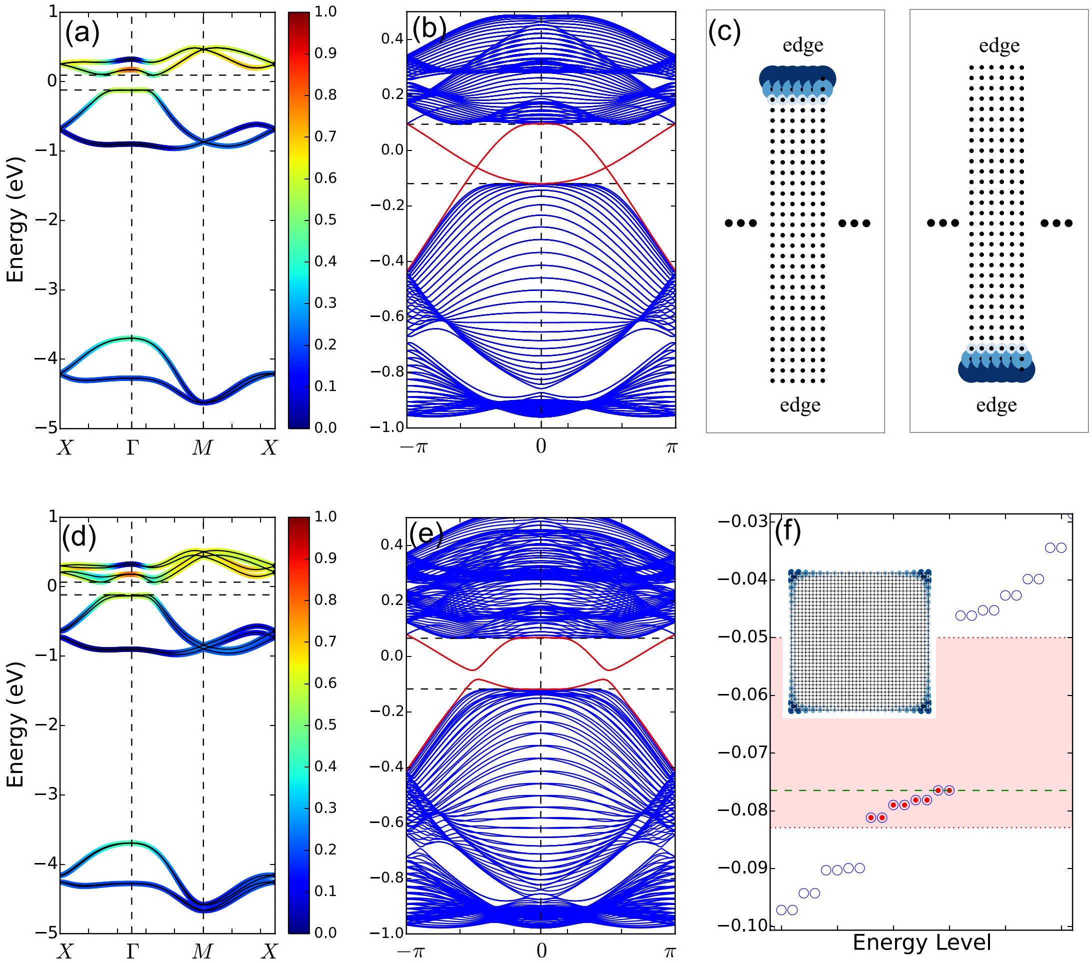

FIG. S1. (a-c) The fragile TCI state in the buckled square lattice. (a) bulk band, (b) gapless edge state in a nanoribbon structure with smooth edges, (c) spacial distribution of edge states. (d-f) The electronic state of the buckled square lattice with a staggered potential. (d) bulk bands, (e) gapped edge state in the same nanoribbon structure, (f) energy levels in disk structure where corner states are marked in red. The inset shows the spacial distribution of corner states.

II. FRAGILE TCI IN BUCKLED SQUARE LATTICE

The bulk band topology discussed in the main text also suggests the coexistence of fragile TCI phase[4,5]. The $\mathcal{S}_4$ symmetry in the buckled square lattice actually pledges sublattice symmetry. The Zak phase $\varphi_{\pm i} = \pi$ represents the electric polarization for a mirror subspace of the 1D system, dictating the presence of edge states for each mirror subspace at an edge where the $\mathcal{M}_d$-invariant k-path has a finite projection. Particularly, degenerate in-gap states appear along the smooth diagonal edge, as shown in Fig. S1(a)-(c). Therefore, the system is a TCI protected by the diagonal mirror symmetry.

However, this gapless edge state will be destroyed by breaking the AB sublattice symmetry with staggered potential. Different on-site energies on AB sites break sublattice symmetry, hence destroy the gapless edge states of the fragile TCI, as shown in Fig. S1(d)-(e). In contrast, the topological corner states survive in the presence of staggered potential as shown in Fig. S1(f), indicating that they are much more robust against such perturbations.

III. ROBUSTNESS OF TOPOLOGICAL CORNER STATES AGAINST STAGGERED POTENTIALS

For comparison, we also considered the effect of the staggered potential, which breaks the $\mathcal{S}_4$ symmetry, in the buckled square lattice shown in Fig. 1 of the main text. The calculated energy spectrum of nanoribbon and nanodisk of the buckled square lattice with staggered potential are presented in Fig. S2. In comparison with Fig. 1(h)-(i) in the main text, the gapped edge bands are no longer degenerate due to the asymmetry of the opposite edges in the

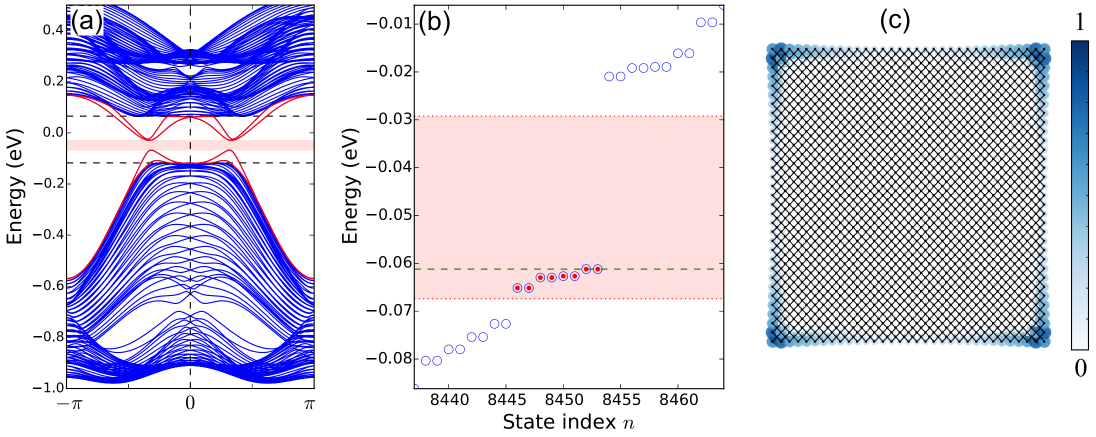

FIG. S2. Energy spectrum of (a) nanribbon and (b) nanodisk of buckled square lattices with staggered potentials. (c) The spacial distribution of corner states.

buckled lattices. However, the energy levels of four pairs of corner states only split slightly and these states remain well localized at corners of the sample, as shown in Fig.Fig. S2(b) and (c), indicating that these topological corner states are robust against staggered potentials.

IV. ROBUSTNESS AGAINST BUCKLING HEIGHT FLUCTUATION

To check the robustness of topological corner states, we calculate the spectrum of buckled square lattice with random buckling height. Specifically, starting from a 30×30 buckled square lattice with fixed buckling heights $h_0 = \pm 0.05a$ (where a is the bond length and $+/-$ is for upwards/downwards buckled atoms), we add a random height δh which is uniformly distributed in the interval $[-0.05a, 0.05a]$ for each atom. Therefore, the atomic heights of upwards and downwards buckled atoms fluctuate in $[0, 0.1a]$ and $[-0.1a, 0]$, respectively. Based on the above setup, an approximate $\mathcal{S}_4$ symmetry is overall preserved. As shown in Fig. 2 of the main text, we found that corner states persist in asymmetric samples with buckling height fluctuation.

V. ROBUSTNESS AGAINST RANDOM IN-PLANE DISPLACEMENT

In addition, we also investigate the effect of in-plane random displacements on the topological corner states. For the buckled square lattice with fixed buckling height, we assign random in-plane atomic displacements $\mathbf{r} = \delta(\cos\theta, \sin\theta)$ for individual atoms, where θ and δ is a random angle and a amplitude uniformly distributed in the interval $[0, 2\pi)$ and $[0, 0.15a)$, respectively. As shown in Fig. S3, topological corner states persist in the presence of in-plane displacements, indicating that the corner states are robust against weak symmetry-breaking perturbations. However, with increasing atomic displacements, the HOTI state is destroyed and topological corner states disappear.

VI. TIGHT-BINDING MODEL PARAMETERS FOR VARIOUS LATTICES WITH DIFFERENT SYMMETRIES

Figure S4-S11 show the bulk and nanoribbon band structure of various lattices without/with (planar/buckled) structural buckling. The tight-binding parameters are provided in the caption of each figures.

VII. DFT CALCULATION OF BUCKLED SB HONEYCOMB LATTICES

Figure S12 shows the TCI state in the planar honeycomb lattice of Sb. Figure S13 shows the QSH states in low-buckled honeycomb structures of Sb.

As shown in Fig. S14, we compare the band structures without and that with SOC for the zigzag nanoribbon of the natively buckled Sb monolayer. The effect of edge saturation of zigzag and armchair nanoribbons are shown in

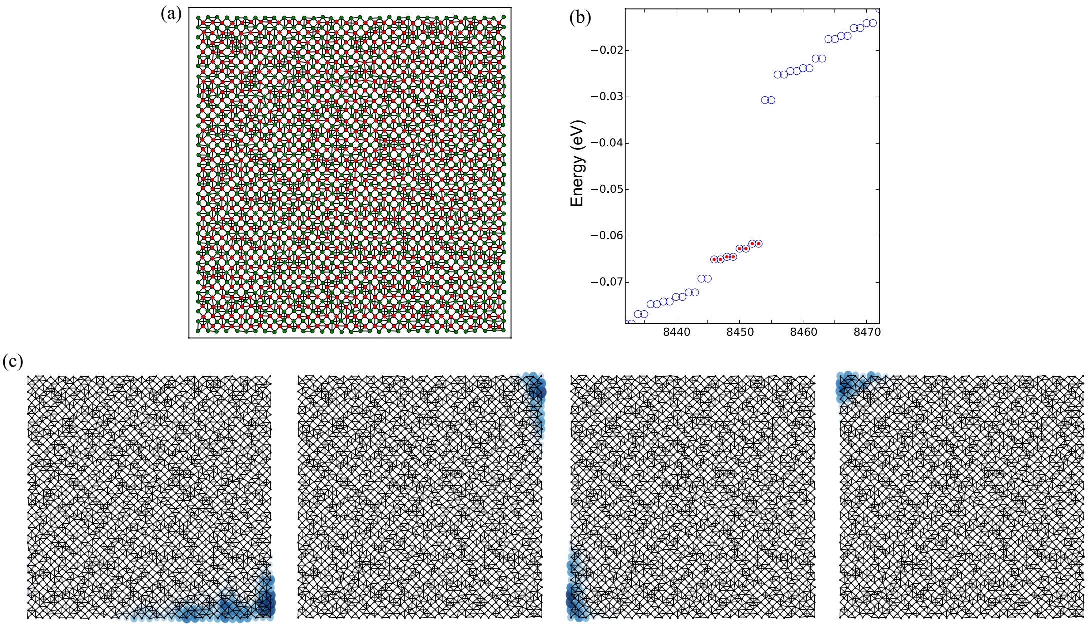

FIG. S3. (a) A buckled square lattice with in-plane random displacements. (b) Energy spectrum of a square disk of the buckled square lattice with in-plane random displacements. The corner states are marked in red. (c) The spacial intensity distribution $|\psi(\mathbf{r})|^2$ of in-gap corner states.

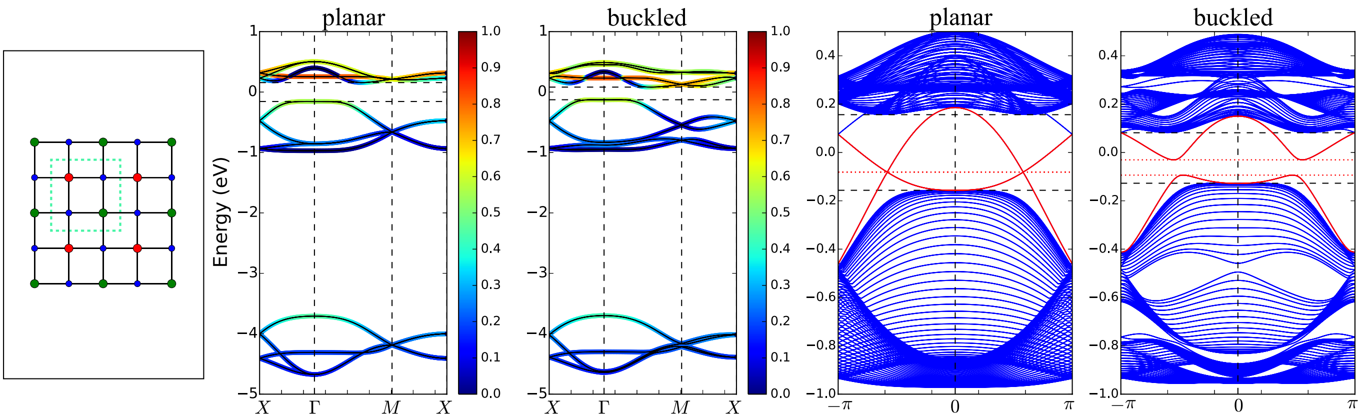

FIG. S4. HOTI in FeTe-like tetrahedral-buckled square lattice. The color (red/green) marks atomic bukling direction (upwards/downwards). The parameters used here are $\epsilon_{x,y} = -1.88, \epsilon_z = -0.88, V_{pp\sigma} = 0.49, V_{pp\pi} = -0.15$, and $\lambda = 1.25$ eV. The buckling height is $h = 0.3a$ with a being the bond length.

Figs. S15 and S16, respectively. Figure S17 shows the energy spectrum of nanodisk of the buckled Sb honeycomb lattice without edge saturation. Because edges are not saturated by H, both edge states (see Fig. S16(a)) and corner states appear near the Fermi level. The inset shows the real-space charge distribution of corner states around the Fermi level.

[1] G. Bir and G. Pikus, Sov. Phys. Solid State **3**, 2221 (1962).
[2] S.-Q. Shen, *Topological insulators*, Vol. 174 (Springer, 2012).
[3] J. Liu, T. H. Hsieh, P. Wei, W. Duan, J. Moodera, and L. Fu, Nat. Mater. **13**, 178 (2014).
[4] M. Ezawa, Phys. Rev. Lett. **121**, 116801 (2018).
[5] Y. Ren, Z. Qiao, and Q. Niu, Phys. Rev. Lett. **124**, 166804 (2020).
[6] C.-H. Hsu, Z.-Q. Huang, C. P. Crisostomo, L.-Z. Yao, F.-C. Chuang, Y.-T. Liu, B. Wang, C.-H. Hsu, C.-C. Lee, H. Lin,

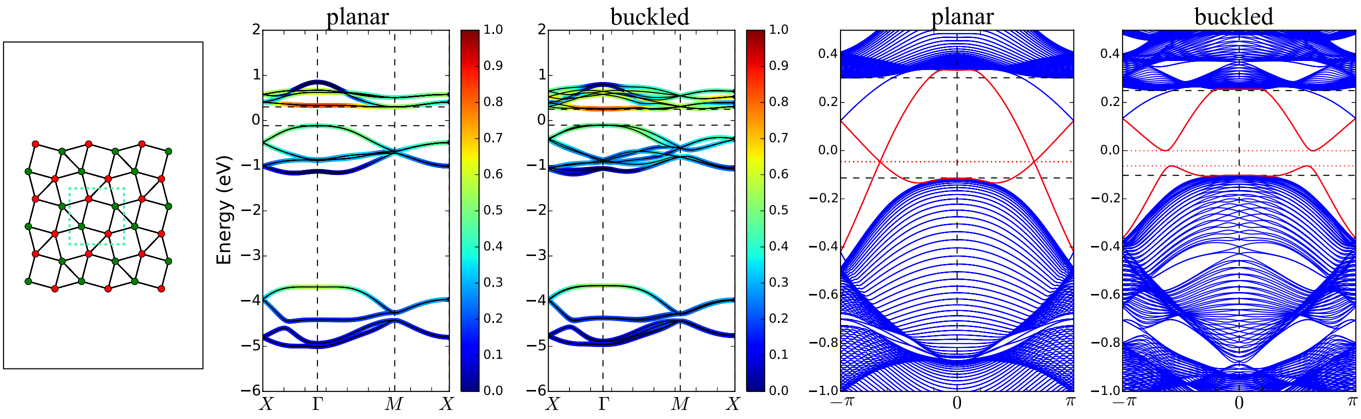

FIG. S5. HOTI in buckled snub square lattice. The color (red/green) marks atomic bukling direction (upwards/downwards). The parameters used here are $\epsilon_{x,y} = -1.88, \epsilon_z = -0.88, V_{pp\sigma} = 0.71, V_{pp\pi} = -0.21$, and $\lambda = 1.25$ eV. The buckling height is $h = 0.31a$ with a being the bond length.

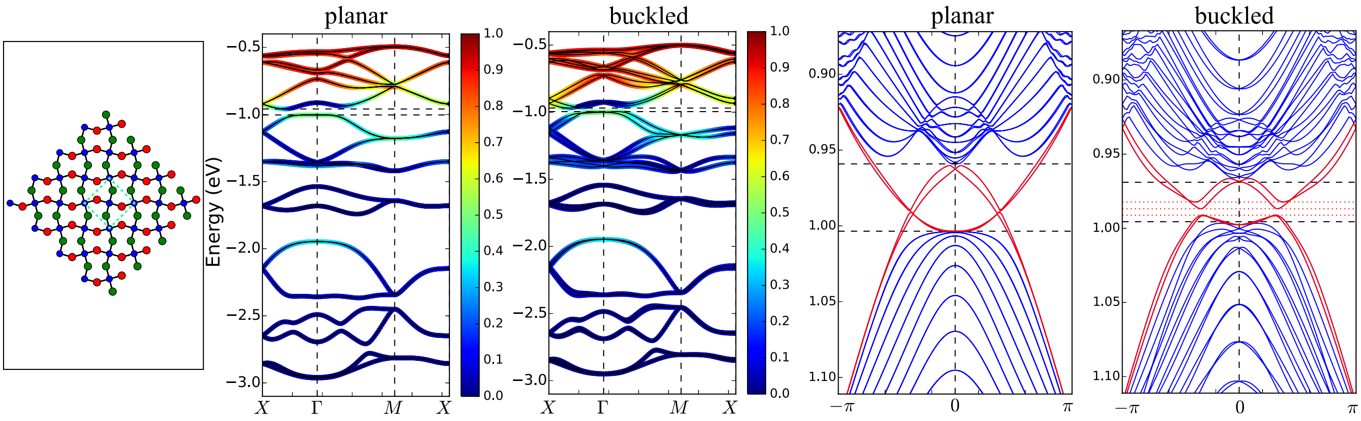

FIG. S6. HOTI in buckled distorted Lieb lattice. The color (red/green) marks atomic bukling direction (upwards/downwards). The parameters used here are $\epsilon_{x,y} = -1.88, \epsilon_z = -0.88, V_{pp\sigma} = 0.41, V_{pp\pi} = -0.12$, and $\lambda = 0.3$ eV. The buckling height is $h = 0.2a$ with a being the bond length.

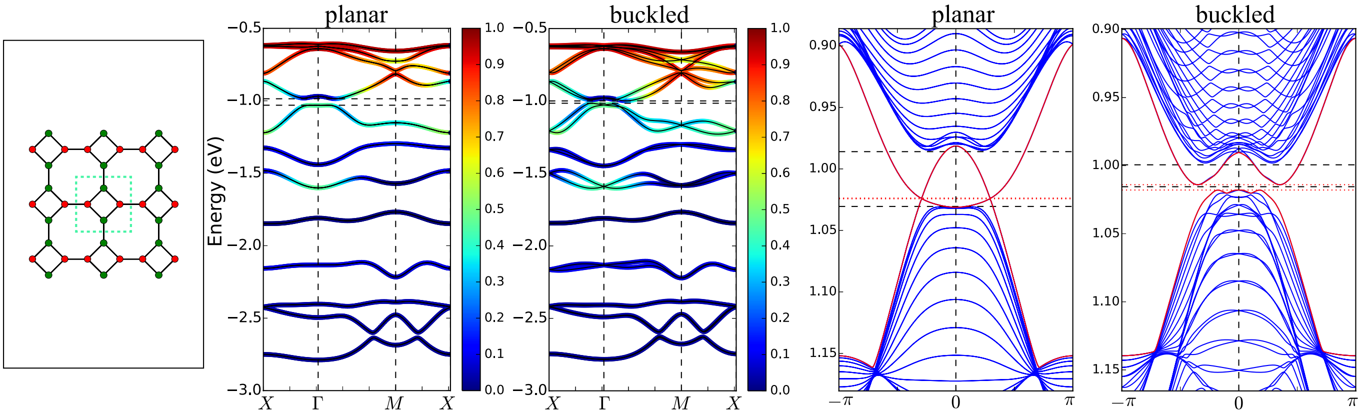

FIG. S7. HOTI in buckled truncated square lattice. The color (red/green) marks atomic bukling direction (upwards/downwards). The parameters used here are $\epsilon_{x,y} = -1.88, \epsilon_z = -0.88, V_{pp\sigma} = 0.36, V_{pp\pi} = -0.11$, and $\lambda = 0.25$ eV. The buckling height is $h = 0.12a$ with a being the bond length.

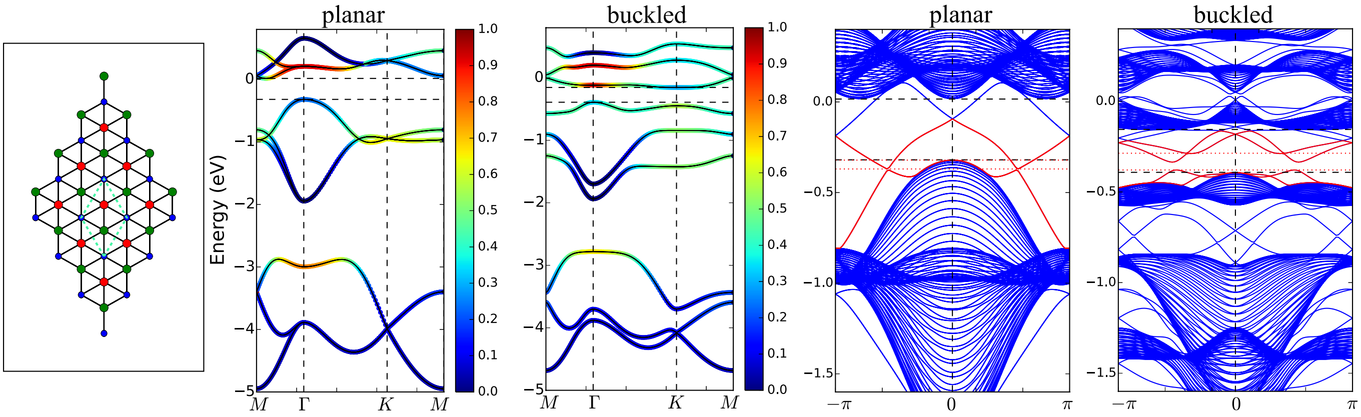

FIG. S8. HOTI in octahedral-buckled trigonal lattice. The color (red/green) marks atomic bukling direction (upwards/downwards). The parameters used here are $\epsilon_{x,y} = -1.88, \epsilon_z = -0.88, V_{pp\sigma} = 0.83, V_{pp\pi} = -0.25$, and $\lambda = 0.8$ eV. The buckling height is $h = 0.35a$ with a being the bond length.

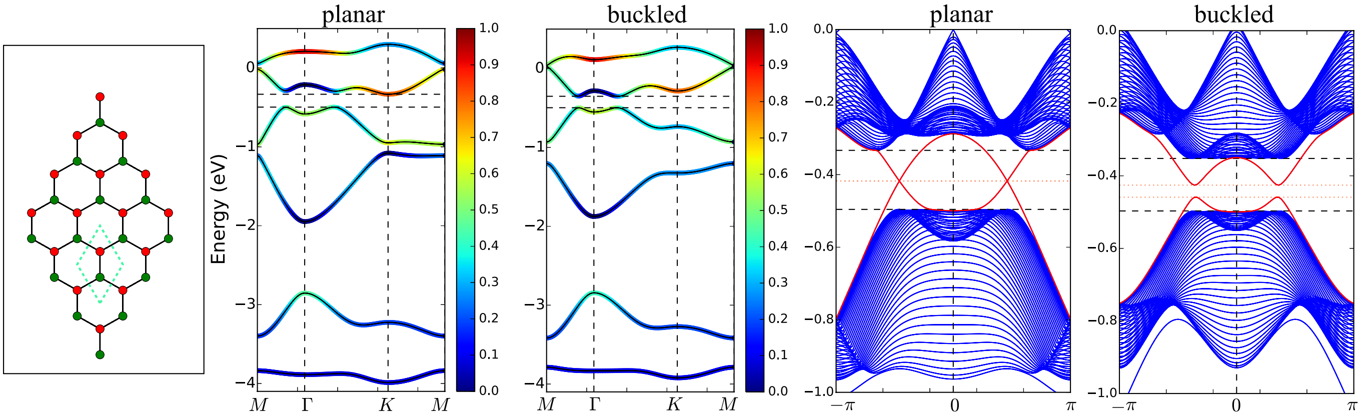

FIG. S9. HOTI in buckled honeycomb lattice. The color (red/green) marks atomic bukling direction (upwards/downwards). The parameters used here are $\epsilon_{x,y} = -1.88, \epsilon_z = -0.88, V_{pp\sigma} = 0.83, V_{pp\pi} = -0.25$, and $\lambda = 0.8$ eV. The buckling height is $h = 0.17a$ with a being the bond length.

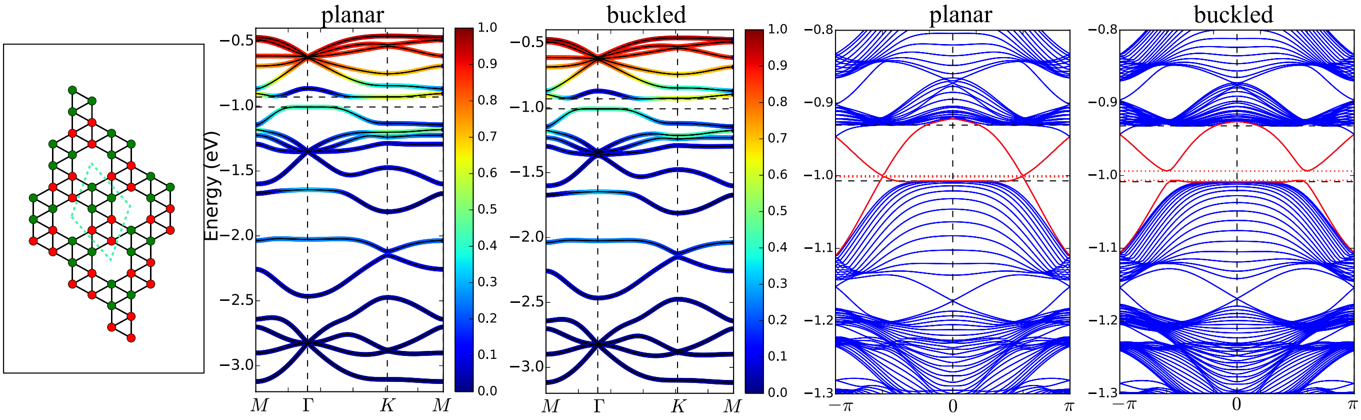

FIG. S10. HOTI in buckled snub hexagonal lattice. The color (red/green) marks atomic bukling direction (upwards/downwards). The parameters used here are $\epsilon_{x,y} = -1.88, \epsilon_z = -0.88, V_{pp\sigma} = 0.40, V_{pp\pi} = -0.12$, and $\lambda = 0.3$ eV. The buckling height is $h = 0.06a$ with a being the bond length.

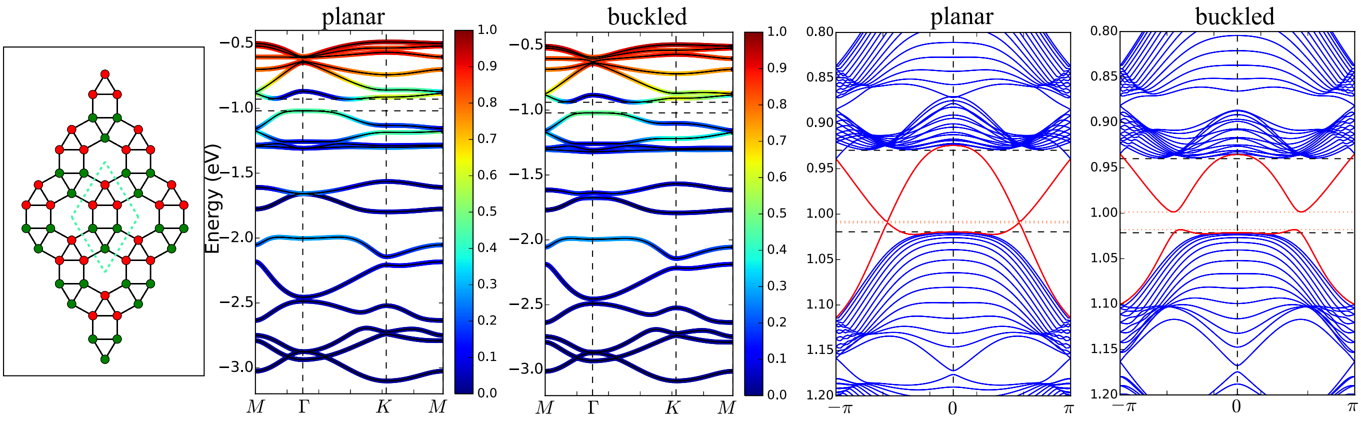

FIG. S11. HOTI in buckled ruby lattice. The color (red/green) marks atomic bukling direction (upwards/downwards). The parameters used here are $\epsilon_{x,y} = -1.88, \epsilon_z = -0.88, V_{pp\sigma} = 0.40, V_{pp\pi} = -0.12$, and $\lambda = 0.3$ eV. The buckling height is $h = 0.27a$ with a being the bond length.

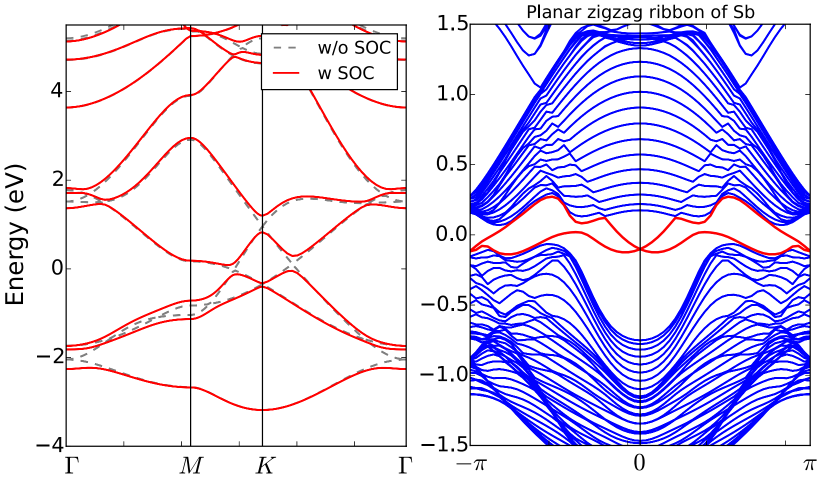

FIG. S12. TCI in the planar honeycomb lattice of Sb. Although the optimized lattice constant is 5.04 Å, to obtain a larger energy gap for the observation of topological edge states, we artificially increase the lattice constant to 5.35 Å, which doesn't change its band topology[6]. (a) Bulk band structure of the planar Sb monolayer without (gray dashed line) and with SOC (red solid line). (b) Band structure of zigzag nanoribbon of the planar Sb monolayer with SOC.

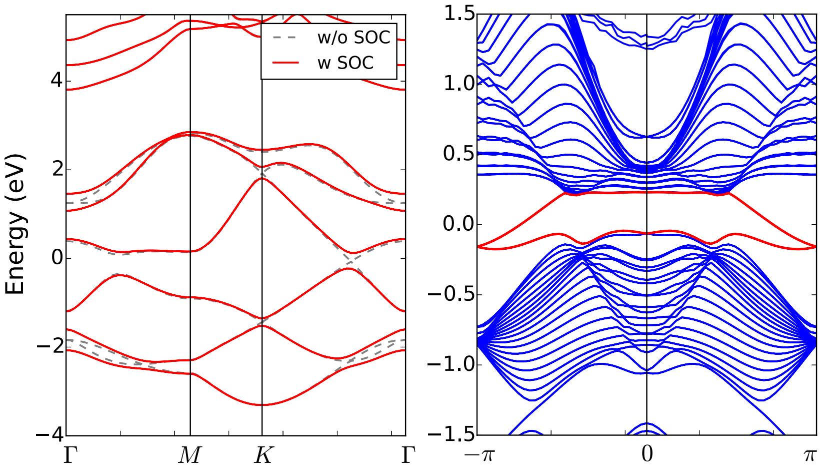

FIG. S13. QSH state in the low-buckled honeycomb lattice of Sb. The lattice constant and the buckling height are set as 5.04 Å and 1.1 Å, respectively. (a) Bulk band structure of the low-buckled Sb monolayer without (gray dashed line) and with SOC (red solid line). (b) Band structure of a zigzag nanoribbon of the low-buckled Sb monolayer with SOC.

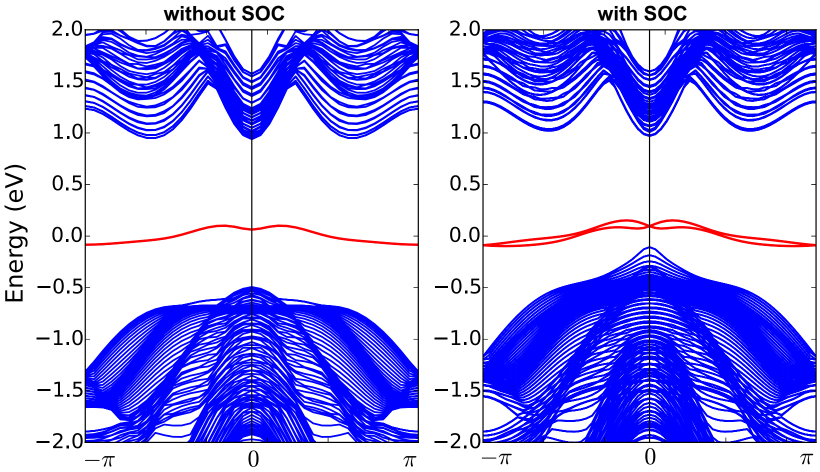

FIG. S14. Band structures of zigzag nanoribbons of the buckled Sb honeycomb lattice. (a) Without SOC, (b) with SOC.

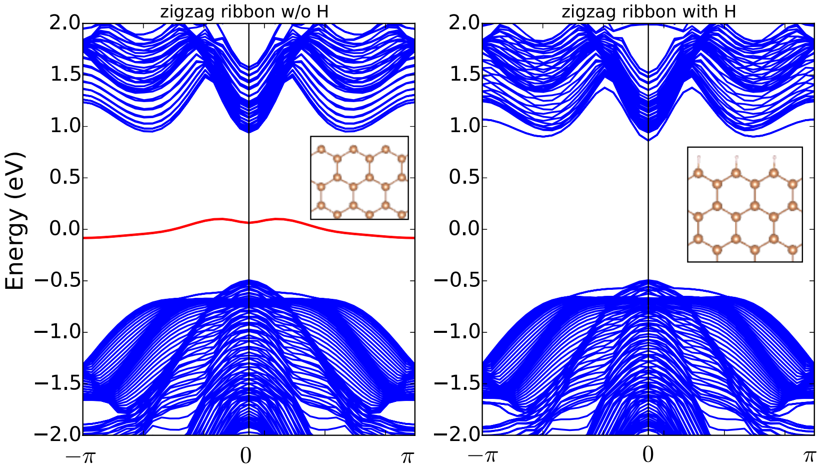

FIG. S15. Band structures of zigzag nanoribbons of the buckled Sb honeycomb lattice without SOC. (a) No edge saturation, (b) Edge saturated by H.

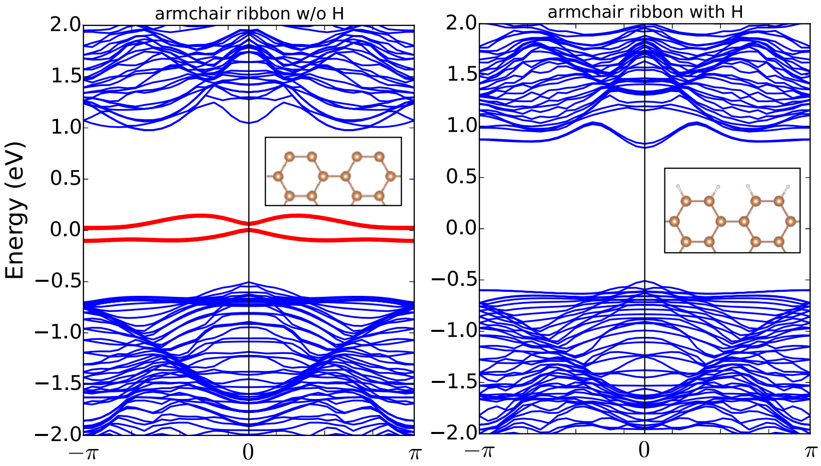

FIG. S16. Band structures of armchair nanoribbons of the buckled Sb honeycomb lattice without SOC. (a) No edge saturation, (b) Edge saturated by H.

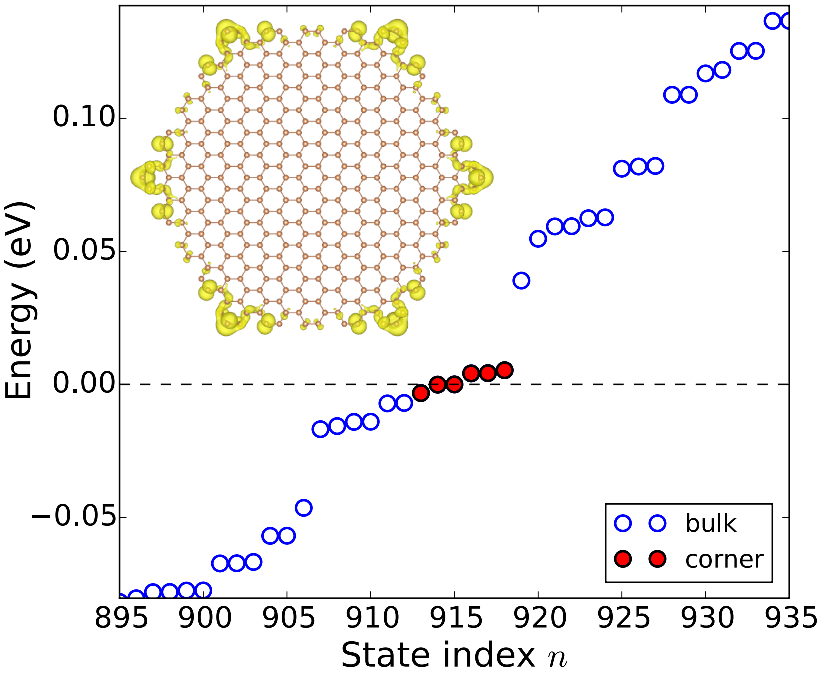

FIG. S17. Energy spectrum of nanodisk of the buckled Sb honeycomb lattice without edge saturation. Because edges are not saturated by H, both edge states (see Fig. S16(a)) and corner states appear near the Fermi level. The inset shows the real-space charge distribution of corner states around the Fermi level.

et al., Sci. Rep. **6**, 18993 (2016).
